# Antiphospholipid syndrome with chronic thromboembolic pulmonary hypertension and coronary artery disease: a case report

**DOI:** 10.1186/s13019-020-01254-4

**Published:** 2020-08-24

**Authors:** Kayo Sugiyama, Shun Suzuki, Nobusato Koizumi, Hitoshi Ogino

**Affiliations:** 1 Department of Cardiac Surgery, Aichi Medical University, 1-1 Yazakokarimata, Nagakute, Aichi 480-1195 Japan; 2 Department of Cardiovascular Surgery, Tokyo Medical University, 6-7-1 Nishishinjuku, Shinjuku-ku, Tokyo, 160-0023 Japan

**Keywords:** Antiphospholipid syndrome, Chronic thromboembolic pulmonary hypertension, Pulmonary endarterectomy, Coronary artery bypass grafting

## Abstract

**Background:**

Antiphospholipid syndrome (APS) is characterized by the production of antiphospholipid antibodies associated with recurrent vascular thrombosis. There have been few reports of combination of chronic thromboembolic pulmonary hypertension (CTEPH) and coronary artery disease in APS, therefore, it is unclear about appropriate treatment strategy.

**Case presentation:**

The patient was a 39 year-old-lady who had been suffering from hypoxia without chest pain. Transthoracic echocardiography showed severe pulmonary hypertension and mild hypokinesis of left ventricular anteroseptal wall. Simultaneously with the diagnosis of CTEPH, coronary angiography revealed severe stenosis of the left anterior descending artery. She underwent pulmonary endarterectomy (PEA) concomitant with coronary artery bypass grafting (CABG) successfully. CABG could be performed concomitantly during rewarming. During perioperative period, she was free from any thromboembolic and bleeding events despite receiving anticoagulant and antiplatelet therapies.

**Conclusions:**

PEA concomitant with coronary artery bypass grafting was feasible for APS patients complicated with CTEPH and coronary artery disease. APS patients with the presence of left ventricular dysfunction should be evaluated for coronary artery disease.

## Background

Antiphospholipid syndrome (APS) is diagnosed based on both clinical criteria and laboratory criteria [1, 2]. The clinical signs include recurrent arterial and venous thrombosis [1]. APS occurs in about 15–50% chronic thromboembolic pulmonary hypertension (CTEPH) patients [3]. Surgical treatment for severe CTEPH with APS is still challenging because the perioperative management may be complicated due to the presence of thrombotic events and bleeding complications [1, 4, 5]. Otherwise there is a risk that coronary artery disease may be overlooked in CTEPH patients with APS. Evaluation of coronary artery disease should be considered in symptomatic APS patients; however, sometimes coronary angiography is necessary even in asymptomatic patients.

Ballon pulmonary angioplasty (BPA) has been known as an effective endovascular treatment; however, its effectiveness is limited to only distal lesions, and PEA is the most appropriate treatment for CTEPH with central lesions [6]. Because in PEA, the patients have to be cooled down to 18 °C [7], rewarming requires more than 1 h. Therefore, concomitant procedures can be usually performed during rewarming.

## Case report

A 39-year-old woman who had been receiving medical treatment for CTEPH for 2 years was referred to our institute for surgical treatment. She had been suffering from hypoxia without chest pain. She had been receiving daily medications including warfarin, riociguat, and eplerenone, and was on home oxygen therapy at 2 L/minute. She had also been diagnosed with Sjögren’s syndrome. She was married, but had never been pregnant. She did not have other risk factors related to atherosclerosis.

**Fig. 1:**
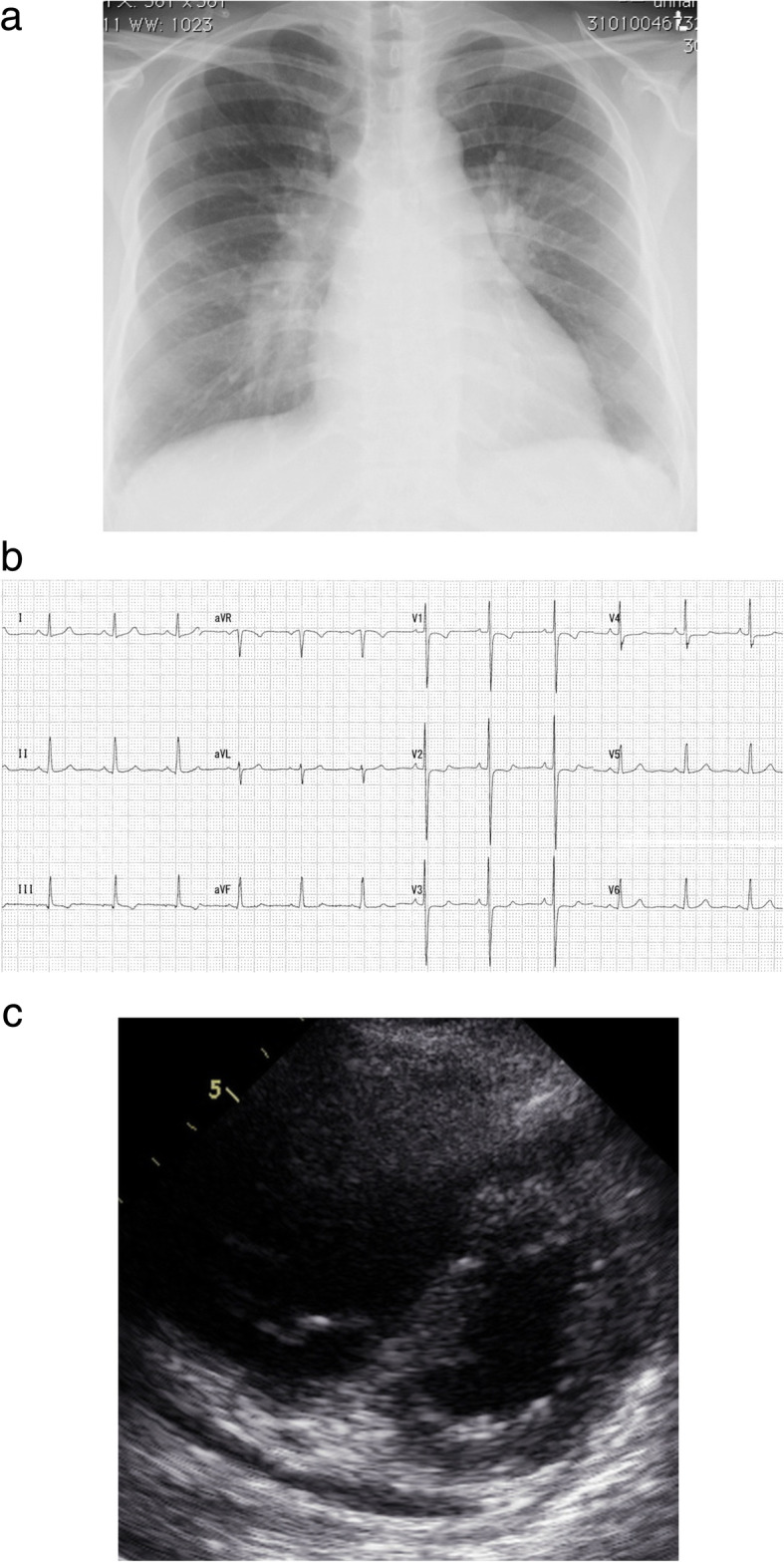
**a**. Preoperative chest radiograph showing cardiomegaly involving the right cavities and notable enlargement of the pulmonary arch. **b**. Preoperative electrocardiogram showing right ventricular hypertrophy and no ischemic changes. **c**. Preoperative echocardiography showing D-shaped compression of left ventricle

**Fig. 2:**
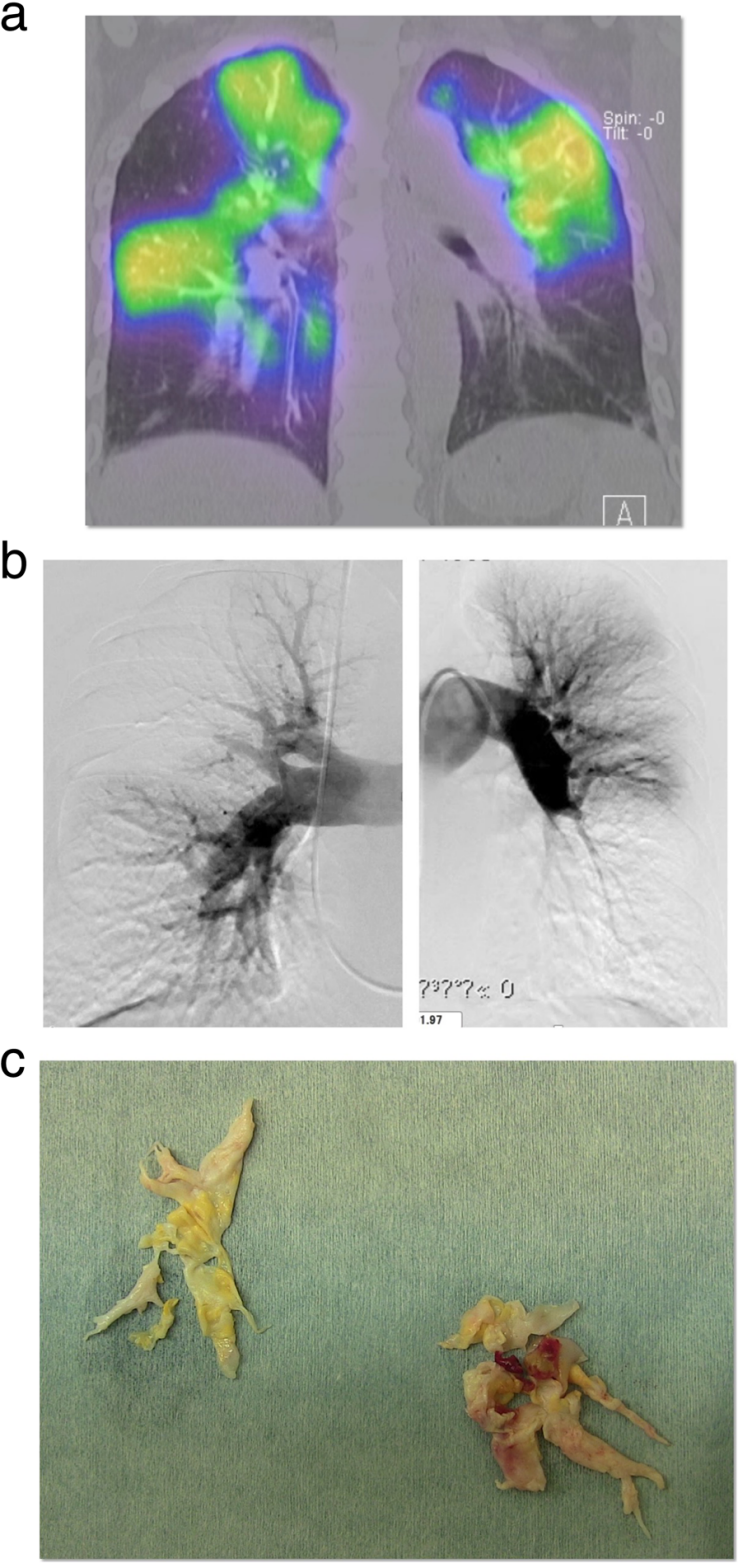
**a**. Preoperative lung perfusion scintigraphy showing multiple segmental defects. **b**. Preoperative pulmonary artery angiograms showing intimal irregularities and abrupt narrowing of both pulmonary arteries. **c**. Resected thromboembolism of both pulmonary arteries

**Fig. 3:**
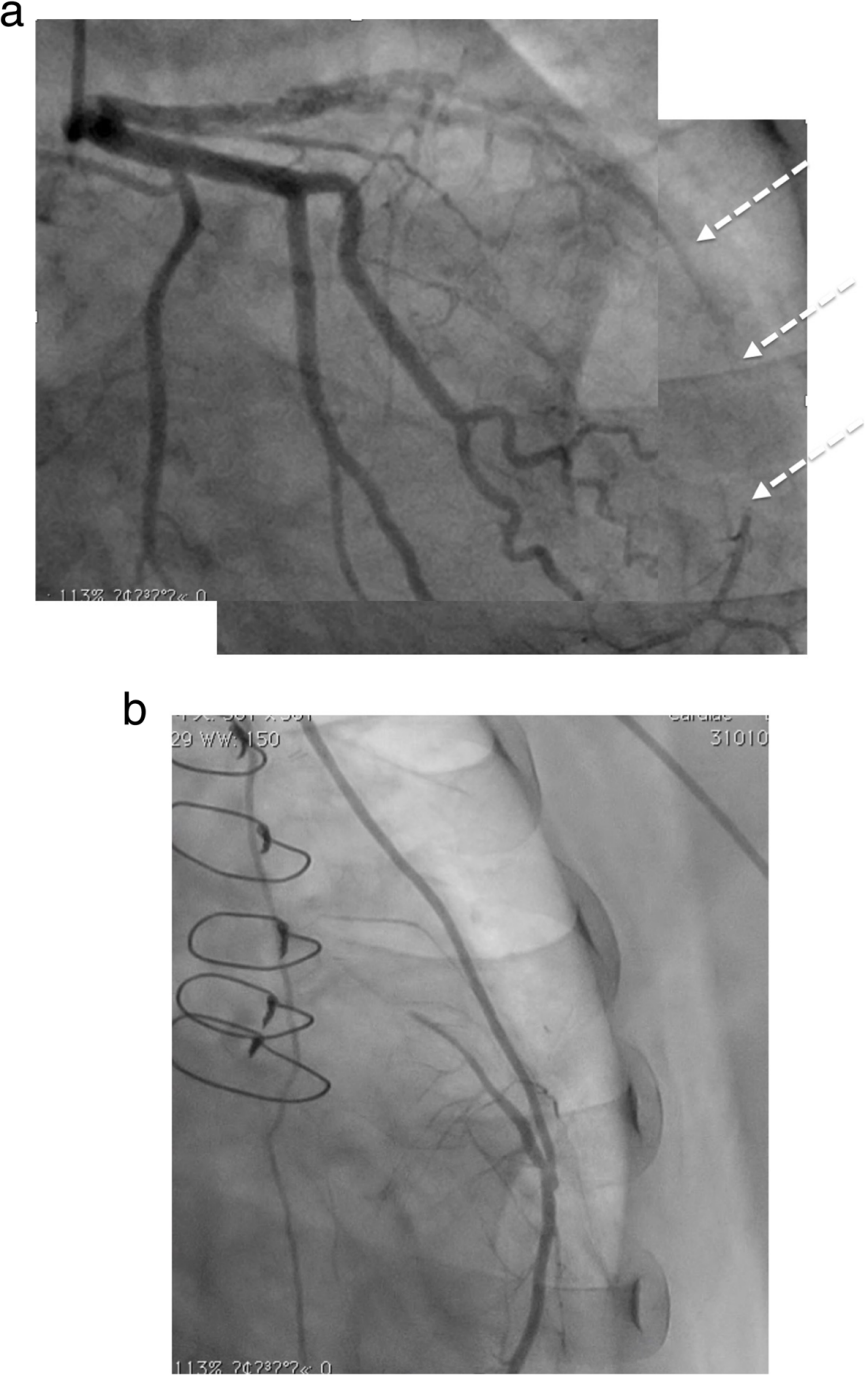
**a**. Preoperative coronary angiography showing severe stenosis in left anterior descending artery (dotted white arrows) **b**. Postoperative coronary angiography showing patent left internal mammary artery to left anterior descending artery

The patient’s preoperative laboratory data were as follows: hemoglobin, 11.5 mg/dL; platelet count, 2.1 × 10^5^/μL; D-dimer, 0.26 μg/mL; prothrombin time -international normalized ratio, 2.0; activated partial thromboplastin time, 68 s; and N-terminal pro-brain natriuretic peptide, 32 pg/mL. Although deficiencies in antithrombin III, protein C and protein S and the hyperhomocysteinemia were not demonstrated, antiphospholipid antibodies were mildly elevated: anticardiolipin antibodies, 43 U/mL; lupus anticoagulant, 1.3 s; and anti-beta2-glycoprotein I antibody, 12 U/mL. The chest radiograph **(**Fig. 1a**)** and chest computed tomography scan showed cardiomegaly involving the right cavities with notable enlargement of the pulmonary arch. The electrocardiogram showed right heart strain and no remarkable ischemic changes **(**Fig. 1b**)**. Echocardiography showed left ventricular compression due to dilated right ventricle **(**Fig. 1c**)** and mild hypokinesis of left ventricular anteroseptal wall. In echocardiography, the right ventricular function was normal and the estimated pulmonary artery pressure was 79 mmHg. Lung perfusion scintigraphy demonstrated multiple perfusion defects in both lungs **(**Fig. 2a**)**. The pulmonary angiogram showed intimal irregularities and abrupt narrowing of both pulmonary arteries **(**Fig. 2b**)**. The coronary angiogram demonstrated severe stenosis in the mid left anterior descending artery **(**Fig. 3a**)**. The rest of the coronary branches showed no significant atherosclerotic changes. The cardiac scintigraphy revealed incomplete reperfusion on the anteroseptal wall. The right heart catheter examination showed severe pulmonary hypertension, with the pulmonary artery pressure, 80/25 mmHg (mean, 45 mmHg) and a pulmonary vascular resistance of 544 dynes/sec/cm^− 5^.

Standard PEA for CTEPH using intermittent circulatory arrest under deep hypothermia and concomitant coronary artery bypass grafting to the left anterior descending artery using a great saphenous vein graft were performed. At surgery, pulmonary artery lesions were approached through a median sternotomy. Cardiopulmonary bypass was established with ascending aortic cannulation and bicaval venous drainage. After left ventricular venting, the patient was cooled down to 18 °C. PEA was performed using a cycle of 15 min of deep hypothermic circulatory arrest followed by 10 min of systemic reperfusion, similar to the techniques established by Jamieson et al. [7]. Endarterectomy specimen was multiple organized thrombi from bilateral pulmonary arteries **(**Fig. 2c**).** During rewarming, coronary artery bypass grafting was performed. The weaning from cardiopulmonary bypass was uneventful and pulmonary hypertension improved to almost within normal range. Strict treatment with anticoagulant and antiplatelet agents was started from the day after surgery. The postoperative course was uneventful and the postoperative examination showed dramatic improvement of her pulmonary hypertension, with a mean pulmonary artery pressure of 15 mmHg and a pulmonary vascular resistance of 207 dynes/sec/cm^− 5^. Echocardiography revealed improvement of left ventricular wall motion. The postoperative coronary angiogram showed a patent bypass graft **(**Fig. 3b**)**. Throughout the postoperative period, she was free from any thromboembolic and bleeding complications. Home oxygen therapy was completely weaned in 1 year, and the patient has remained free from any cardiovascular events for 3 years after surgery.

## Discussion

The antiphospholipid syndrome (APS) is an autoimmune disorder of acquired hypercoagulability characterized by recurrent vascular thrombosis. APS is diagnosed based on both clinical criteria and laboratory criteria [1, 2]. The clinical signs include recurrent arterial and venous thrombosis such as myocardial infarction and chronic thromboembolic pulmonary hypertension (CTEPH) [1]. According to the results of several cohort studies, APS occurs in about 15–50% CTEPH patients [3]. Pulmonary endarterectomy (PEA) is the most appropriate treatment for CTEPH with central lesions, however, there have been few reports describing detailed results of PEA in CTEPH patients with APS.

Furthermore, in patients with APS, the management of anticoagulation becomes complex because of hypercoagulation and bleeding tendency [1, 4, 5]. Neurological complications including stroke and severe thrombocytopenia were more common after PEA in patients with APS [1]. In APS patients prior to main surgery, management of platelet count is problematic [4]. In APS patients, prevalence of thrombocytopenia was found [5]. Therefore, the anticoagulant therapy after PEA for CTEPH patients with APS should be introduced carefully and strictly, however, it is also necessary to pay attention to the bleeding tendency. Further studies regarding the impact of APS on outcomes of PEA are warranted.

In PEA, after establishment of cardiopulmonary bypass, the patient was cooled down to 18 °C and thromboendarterectomy was performed using a cycle of 15 min of deep hypothermic circulatory arrest followed by 10 min of systemic reperfusion [7]. Therefore, bleeding complications can be more seriously in PEA than other cardiac surgeries because of inhibition in coagulation ability due to deep hypothermia. Moreover, PEA sometimes develops critical endobronchial bleeding, therefore, it is necessary to pay attention to the bleeding tendency in CTEPH patients with APS.

Recently, balloon pulmonary angioplasty (BPA) has been performed more frequently in patients with severe pulmonary hypertension due to distal lesions. BPA has been known as an effective endovascular treatment; however, its effectiveness is limited to only distal lesions, and PEA is the most appropriate treatment for CTEPH with central lesions [6]. In the present case, only PEA was judged to be effective in treating severe pulmonary hypertension.

Because rewarming requires more than 1 h, other procedures usually could be performed. In the present case, CABG could be performed concomitantly during rewarming. The reason why we chose great saphenous vein is that saphenous vein graft is more suitable than other artery grafts for gaining quick and sufficient blood flow in ischemic coronary arteries. Furthermore, the systematic approach to saphenous vein grafts has recently developed and mitigated the problem of impaired long-term patency in saphenous vein grafts [8]. We always harvest saphenous vein grafts carefully in consideration with harvesting technique and handling, graft storage and preservation, anastomosis technique and pharmacotherapy after CABG.

2Cardiac involvement in APS may present as heart valve disease affecting approximately a third of patients, or less frequently as intracardial thrombosis, pulmonary hypertension, right or left ventricular dysfunction, micro-vascular thrombosis, or coronary artery disease [9]. In a European cohort of 1000 patients with APS, myocardial infarction was diagnosed in 5.5% of APS patients [10]. In a recent systematic review, the estimated frequency of antiphospholipid antibodies among patients with myocardial infarction was 11% [11]. In accordance with these reports, coronary angiography should be considered in every APS patient with chest symptoms. Moreover, coronary angiography should be considered even in an asymptomatic APS patient if the case shows slight left ventricular dysfunction. Especially in CTEPH patients, because their daily lives are limited due to hypoxia, it is difficult for their chest symptoms to appear. In CTEPH patients, it is mandatory to keep in mind that APS is associated with coronary artery disease.

## Conclusions

Although the perioperative management may be complicated due to the presence of thrombotic events and bleeding complications, PEA concomitant with CABG was feasible in a CTEPH case with APS. CTEPH patients with APS presenting left ventricular dysfunction should be evaluated for coronary artery disease.

## Data Availability

Not applicable.

## Abbreviations

APS: Antiphospholipid syndrome
BPA: Balloon pulmonary angioplasty
CABG: Coronary artery bypass grafting
CTEPH: Chronic thromboembolic pulmonary hypertension
PEA: Pulmonary endarterectomy
